# Bioactive Peptides from Barnacles and Their Potential for Antifouling Development

**DOI:** 10.3390/md21090480

**Published:** 2023-08-30

**Authors:** Xuan Liu, Hui Jin, Gaochi Xu, Ren Lai, Aili Wang

**Affiliations:** 1 Center for Evolution and Conservation Biology, Southern Marine Science and Engineering Guangdong Laboratory (Guangzhou), Guangzhou 511458, China; liuxuan@gmlab.ac.cn (X.L.); jinhui0127@126.com (H.J.); xugaochi@gmlab.ac.cn (G.X.); rlai@mail.kiz.ac.cn (R.L.); 2 Key Laboratory of Bioactive Peptides of Yunnan Province, KIZ-CUHK Joint Laboratory of Bioresources and Molecular Research in Common Diseases, National Resource Center for Non-Human Primates, Kunming Primate Research Center, National Research Facility for Phenotypic & Genetic Analysis of Model Animals (Primate Facility), Sino-African Joint Research Center and Engineering Laboratory of Peptides, Kunming Institute of Zoology, Kunming 650107, China; 3 University of Chinese Academy of Sciences, Beijing 100049, China

**Keywords:** antifouling, barnacle-derived peptides, barnacles, biofouling, natural products

## Abstract

Barnacles, a prevalent fouler organism in intertidal zones, has long been a source of annoyance due to significant economic losses and ecological impacts. Numerous antifouling approaches have been explored, including extensive research on antifouling chemicals. However, the excessive utilization of small-molecule chemicals appears to give rise to novel environmental concerns. Therefore, it is imperative to develop new strategies. Barnacles exhibit appropriate responses to environmental challenges with complex physiological processes and unique sensory systems. Given the assumed crucial role of bioactive peptides, an increasing number of peptides with diverse activities are being discovered in barnacles. Fouling-related processes have been identified as potential targets for antifouling strategies. In this paper, we present a comprehensive review of peptides derived from barnacles, aiming to underscore their significant potential in the quest for innovative solutions in biofouling prevention and drug discovery.

## 1. Introduction

The phenomenon of biofouling, which refers to the adhesion of undesired organisms onto solid surfaces, poses a global challenge, entailing significant economic and environmental consequences. As recorded, the financial costs associated with biofouling control have ranged from USD 1.5 to 3 billion per year [1,2]. The scientific community has dedicated significant efforts to exploring suitable strategies for inhibiting the fouling process, with a particular focus on barnacles [3]. As one of the largest macrofouling organisms globally, barnacles have limited natural predators and are frequently employed as target model species in antifouling research. Around 400 natural products derived from marine microorganisms, algae, and marine invertebrates have been documented for their antifouling activity. However, the majority of antifouling agents targeting macrofouling were predominantly composed of small-molecule chemicals and exhibited hazardous properties [4,5,6]. Although certain small-molecule chemicals, such as now-prohibited tributyltin, exhibit favorable performance characteristics, only a limited number have been successfully commercialized and deployed in marine environments. There is a great need to explore more effective, environmentally friendly antifouling agents, particularly those targeting barnacles. Peptides, which play a crucial role in the physiological processes of barnacles [7], could be key targets and promising candidates for screening antifouling agents.

Peptides, consisting of amino acids connected by peptide bonds, have been reported to play a crucial role in the physiological process of barnacles, particularly in relation to their neurosystem and settlement mechanisms [8]. Moreover, peptides serve as excellent signals in marine ecosystems and offer numerous advantages over smaller information molecules like amino acids. This provides an opportunity for the screening of antifouling agents when targeting internal functional peptides in relation to their production processes. Numerous bioactive peptides, which play an essential role in the neurosystem, hormone regulation, cell growth, and reproduction, have been explored [9,10] for drugs and industries with great advantages, including high efficiency, safety, and higher selectivity [11,12]. These characteristics also render peptides promising candidates for antifouling applications. Moreover, barnacles can serve as a rich source of peptides, which are commonly present as bioactive compounds within their structures. Bioinformatic analysis revealed the presence of additional unexplored peptide sequences. Dominguez-Perez’s team found over 11,755 peptide sequences from *Pollicipes pollicipes*, although their separate identification was not conducted [13]. Currently, the majority of barnacle-derived peptides are neuropeptides, as barnacles have demonstrated unique mechanisms for determining their exploratory stage and settlement status, primarily relying on sensory mechanoreceptive and chemoreceptive signals [14]. The physiological function of peptides and in vivo chemical reactions in barnacles, such as larvae development, settlement, intercommunication, and intracommunication, remain enigmatic. There are numerous unexplored barnacle-derived peptides.

Barnacle-derived peptides are a promising yet overlooked source for screening antifouling agents. Approximately 71% of the Earth’s surface is encompassed by oceans, harboring a plethora of bioactive substances with rare and distinctive chemical characteristics derived from their intricate ecological milieu. For instance, sponges possess the capability to synthesize diverse secondary metabolites as a defense mechanism [15]. It has been reported that sponge *Geodia barretti* could produce antifouling cyclopeptide with an EC_50_ value of 15 nM [16,17]. With their gregarious nature, barnacles exhibit a preference for aged fouling sites and are believed to inhibit the growth of other fouling species. These chemical defenses not only manifest at the macroscopic level but also operate within the internal environment of barnacles. The production of prostaglandins could be influenced by feeding and environmental factors, subsequently impacting the reproductive processes of barnacles [18]. Regulating the production and efficacy of keystone peptides could potentially constitute a groundbreaking advancement in biofouling prevention, with numerous antibiofilm peptides having been extensively investigated and recognized as environmentally benign antifouling agents. Post-settlement *A. amphitrite* effectively eradicated the bacterial biofilm using a fluid containing reactive oxygen species (ROS) [8]. Barnacles, commonly employed as a modal organism for screening antifouling compounds, hold potential as a gateway for the targeted validation and screening of antifouling agents.

There is a growing recognition among researchers that barnacle-derived peptides hold significant potential as a promising target and source of bioinformatically based antifouling compounds. Barnacles not only act as fouling organisms but also serve as environmental stressors. The physiological and behavioral adaptability of barnacles enables their utilization as model organisms in various environmental research, including studies on climate change and mental monitoring [19]. These examples demonstrate the significant potential of exploring new functional peptides. Unlike conventional extraction methods, the contemporary approach for investigating natural products involves genome mining based on machine learning, which integrates homology searching, active peptide characterization, and enzymatic hydrolysis strategies [20,21,22]. Barnacle proteomics analysis has revealed the presence of numerous functional proteins [19]. Interestingly, bioinformatic analysis revealed that the metabolite and pathways of barnacles, particularly during the sensing, reproduction, and settlement stages, have a striking similarity with insects undergoing molting processes [23]. This discovery provides a novel avenue for barnacle research, highlighting the intricate nature of peptide-mediated processes and emphasizing the potential insights that could be gained from exploring insect-derived peptides. Although numerous peptides have been investigated using bioinformatic approaches, the majority of them lack comprehensive validation. A comprehensive understanding of the intricate functionalities of peptides is of the utmost importance. A more profound elucidation of the mechanism in barnacles could facilitate the development of novel antifouling strategies.

Given the alarming state of biofouling, it is imperative and opportune to explore novel potential antifouling strategies with reduced toxicity. This review provides a comprehensive summary of barnacle-related peptides categorized according to different physiological processes, offering valuable insights for future investigations into antifouling approaches. Specifically, this review highlights: (i) an overview of various types of barnacle-derived peptides based on physiological roles; (ii) the involvement of peptides in the biofouling process and their functions; and (iii) the potential utility and prospects for further research development in utilizing barnacle-derived peptides. While acknowledging that this review might not be exhaustive, it presents compelling evidence supporting the significance of barnacle-derived peptides in barnacles’ physiological function and their promising applications.

## 2. Barnacles–Smart Macrofouler

### 2.1. The Structure of Barnacles

Barnacles exhibit a complex life cycle consisting of six stages in the planktonic and the sessile phases. The shifting of these stages is highly sensitive to environmental factors, making them potential targets for antifouling agents that can inhibit dynamic variation. The structure of barnacles, particularly the sensory organs, has aroused considerable interest among researchers and scholars. The sensing process of cyprids has been found to be associated with their attachment organs, namely the two attachment discs on the antennules (see Liang et al., 2019 for review) [8]. However, a more detailed dissection is required due to the division of labor and cooperation among organs in many functional physiological processes. Nevertheless, in ripened individuals, some adhesive-secreting cells adhere to the ovary, making it difficult to separate them [13]. Additionally, certain glands, such as cement glands, have been reported to degenerate during molting, further complicating the dissection process. Previous studies by Gallus involved studying the entire soft tissue immediately after its removal from the shell for subsequent bioinformatic research [24] but yielded ambiguous results. Moreover, contamination from bacteria poses challenges in achieving accurate separation and drawing the correct conclusions. For instance, it has been suggested that bacterial activity could contribute to barnacle settlement pheromone’s native serine protease activity [25]. Given that single-cell sequencing is widely employed nowadays, obtaining high-quality clean samples from barnacles becomes crucial [26]. To accomplish this goal effectively, compiling and referencing additional studies is beneficial.

### 2.2. The Role of Barnacle in Biofouling

Barnacles are a globally renowned macrofouler and are recognized as common vector species [27]. They exhibit a ubiquitous distribution within the intertidal zone. Biofouling is a dynamic and intricate process that can be categorized into two components, namely microfouling and macrofouling. In greater detail, biofouling undergoes four stages: (1) the adsorption of organic particles, (2) colonization by primary organisms, (3) the settlement of invertebrates, and (4) the establishment of a complex macroscopic fouling community [28]. Within aquatic environments, microorganisms such as bacteria and algae rapidly attach to surfaces within minutes, leading to biofilm formation until the larvae explore the surface. Ultimately, a diverse macroscopic fouling community is established following the settlement of macroalgal zoospores and invertebrate larvae [28]. In this process, barnacles function as integral components of the macroorganism and establish connections with other fouling organisms. The chemical defenses between these organisms represent a valuable source of antifouling agents, inspiring the further development of antifouling strategies. For instance, certain bacterial species that are capable of forming biofilms have been demonstrated to inhibit the settlement of barnacle larvae [29], and this inhibitory effect has been reciprocated [30]. Given that barnacles possess the ability to cleanse biofilms and engage in communication [8], they serve as a reservoir for anti-microfouling compounds. Y-organs, which are molting glands found in decapod crustaceans, provide animals with flexibility when controlling their molting time. While previous studies have shed light on the functionality of Y-organs in crustaceans [31], there is limited documentation regarding barnacle molting glands specifically associated with the molting process [32]. Understanding the settlement mechanisms of barnacles is closely intertwined with comprehending the biofouling process; thus, investigating the mechanism of this settlement could aid in identifying potential targets for antifouling interventions.

### 2.3. The Settlement and the Molting Processes of Barnacles

A deeper understanding of the molecular mechanism underlying molting and settlement is crucial for identifying novel antifouling targets in barnacles. The settlement process is intricately linked to the fouling substrate, such as the biofilm, with its composition potentially exerting positive or negative effects on the settlement. Additionally, certain barnacle organisms exhibit a preference for pristine surfaces [33,34,35]. It has been postulated that the nervous system plays a pivotal role in controlling the settlement process, whereby larvae are primed for settling once their nervous system reaches full development. Choosing an inappropriate substrate may also result in larval mortality. Perceived cues are believed to be mediated through specific receptors located on a larval sensory organ, with signals transmitted via both nervous and cellular signal transduction systems. Notably, arthropodin, an inducer found in the adult shell of *Balanus balanoides*, has been proposed to participate in the gregarious settlement and represents the first peptide-like signaling molecule discovered [25]. In subsequent investigations, it was found that the larvae exhibited a discernment between conspecific SIPC and those belonging to different species. Based on transcriptome analysis, the MARK cascade, blood coagulation cascade, and Wnt signaling were identified as pivotal regulators in larval development, especially driving the transition to the settlement stage [36]. From a bioactive compound perspective, it has been well-documented that barnacle metamorphosis is regulated via hormones such as methyl farnesoate (sesquiterpenoid) and receptors with structurally comparable features to the insect juvenile hormone [23,37], which exhibit analogous structures compared to those found in insects. Despite not being peptides, the control of methyl farnesoate is intricately linked to neuropeptides [38]. This finding offers a novel perspective on the research methodologies concerning barnacles, suggesting that insights into the molting process and insect pathway could enhance our understanding of these organisms. The settlement and molting processes of barnacles were found to be interconnected yet distinct phenomena. For example, protein kinase C, a family of protein kinase enzymes, was reported to be involved in larval metamorphosis rather than the actual settlement process of *B. amphitrite* [39]. There is compelling evidence to suggest that the process of adhesion occurs in a cyclic, multi-step manner synchronized with the molt cycles [40] and exhibits sensitivity to various environmental conditions and stressors [36,41]. In this study, we discussed these two processes separately, introducing the corresponding peptides in distinct sections.

Feeding is a crucial physiological activity that plays a pivotal role in preventing obesity and metabolic diseases by regulating metabolism. However, the molecular mechanisms underlying the cessation of feeding during the cyprid period in barnacles remain unknown [42]. FMRFamid-like peptides have been identified in *B. amphitrite* and are believed to be essential to digestive processes [43], yet their functional principles have been poorly elucidated. Although several behavioral experiments, including observations of larval swimming behaviors, settlement experiments, and color experiments, have been conducted for validation, there has been limited investigation into appetitive behavior and food consumption—an area extensively studied in fruit fly research but largely unexplored in barnacle research. Since the existence of a non-feeding cyprid stage during the planktonic phase of barnacle life stages has been identified, exploring feeding behavior may offer new insights into inhibiting barnacle settlement. Furthermore, energy levels are closely associated with elevated lipid levels, which could serve as potential indicators for further study and as targets for intervention.

In this section, we aimed to emphasize the significant potential of barnacle-derived peptides due to their close association with the biofouling process. These peptides could serve as promising targets for inhibiting the barnacle settlement or disrupting the fouling process in other organisms via intra- and inter-communication mechanisms. Enhancing our understanding of barnacle-derived peptides and their roles in biofouling could contribute to exploring novel antifouling strategies.

## 3. The Role of Barnacle-Derived Peptides in Biofouling

### 3.1. Neuropeptides—The Regulator

Neuropeptides, bioactive peptides present in the nervous system, play a crucial role as major regulators of physiological processes during barnacle development stages, including growth, reproduction, settlement, environment sensing, molting and cuticle hardening [44], feeding behavior, adaptation to external factors (stress regulation [45] and temperature fluctuation [46]), protein degradation and cell–cell interactions [47]. Crustaceans with a relatively simple neurosystem serve as an excellent model for studying these phenomena. However, recent studies have indicated that neuropeptides in barnacles are not solely produced by the brain or central nervous system [48] but are also expressed in non-neuronal tissues like compound eyes [45]. For example, the syn cerebrum of the cypris exhibits a remarkable complexity involving structures such as frontal filaments, nauplius eyes, compound eyes, and antennules [49]. Though the correlations between different neuropeptides were tested widely, their precise cellular signaling mechanisms remained elusive [50]. These findings collectively indicate that the working principle of neuropeptides can be complex even in a simple system.

Biofouling is a dynamic and complex process, and many parts are mainly controlled by neuropeptides, such as biofilm recognition [47], the settlement decision, and the swimming depth [51]. The pigment dispersing hormone (PDH) was initially isolated from the eyestalks of the shrimp *Pandalus borealis* as a light-adapting hormone and was then also discovered in the barnacle [52]. PDH signaling likely involves adenylate cyclase, cAMP, and protein kinase A, while its localization has been observed in the protocerebrum with a highly intricate internal organization of neuropil regions and tracts consisting of the optic ganglia [53].

The settlement of barnacles was assumed to be regulated by a specialized sensory-neurosecretory system [54], which facilitated decision-making processes. The selection of a suitable substratum for the permanent attachment of competent cyprid relies on their sensitive responses to both the physical and chemical characteristics of the environment, as well as conspecific biogenic cues [55]. However, the underlying mechanisms of this system remain unknown. Compared to the adults, the nerve development in barnacle larvae appears more intriguing and diverse. Previous studies have demonstrated the regulation of PDH, SIFamide, calcitonin, and B-type allatostatin, tachykinin-related peptides in both cyprids and juveniles [7]. FMRFamide (Phe-Met-Arg-Phe-amide, FMRFa), belonging to a family of brain-gut peptides, is widely distributed in the nervous system of the invertebrate [56], and almost 50 FMRFamide IR neurons were identified in *B. amphitrite* [24]. The function of FMRFamide-like peptide IR neurons in the cyprid stage differed from that observed in adult barnacles. In adults, over 50 FMRFamid-like peptides IR were found within the ventral ganglion, while only two of them were involved in rhythmic muscular contractions of the limbs [57]. It is common for peptides to be discovered in barnacles while their exact functions remain unknown, such as the prothoracicotropic hormone [23]. The identification and characterization of the inhibitors and excitants for these functional peptides could provide novel antifouling strategies.

Barnacles, classified as arthropods, are closely related to other crustaceans. However, certain proteins and peptides found in barnacles have been observed to play similar roles in insects [58]. Bioinformatics data further support this interesting relationship; Yan’s work has revealed that some neuropeptides of *B. amphitrite* exhibit a closer affinity with insects than decapods [7]. The FGLamide allatostatin family is widely distributed among insects and crustaceans and has been identified as an inhibitor of juvenile hormone biosynthesis [3,59]. Allatostatins are pleiotropic neuropeptides that have also been widely discovered in insects and crustaceans. They primarily serve two functions: inhibiting juvenile hormone synthesis and reducing food intake. Three types of allatostatins were found in barnacles using meta-transcriptomics and were further verified with a qPCR. A-type allatostatin, regarded as an emerging hormone related to the nutrient state, could accelerate the use of lipids, regulate metabolism and feed decisions, and play an important role in cyprid: the non-feeding stage [60]. B-type allatostatin, also known as a myo-inhibiting peptide, is implicated in controlling visceral muscle contractions, the regulation of metabolism, feeding, and sleep. There is evidence showing that B-type allatostatin is involved in the immune system [61]. C-type allatostatin, characterized by a conserved unblocked PISCF motif at the C-terminus, is separately regulated by the known C-type allatostatin gene [62]. C-type allatostatin has been demonstrated to modulate the neuromuscular system and has been distributed in the stomatogastric nervous system for crabs [63]. Liu et al. found that C-type allatostatin could inhibit vitellogenin absorption and oocyte growth [62]. However, the exact functions of C-type allatostatins in barnacles remain to be further explored. The red pigment-concentrating hormone (pGlu-Leu-Asn-Phe-Ser-Pro-Gly-Trp -amide, localizationRPCH) is widely found in crustaceans and insects but has little record in the barnacle. The absence of RPCH is surprising, and Webster put forward the hypothesis that PDH-like immunoreactive (PDHLI) neurons could override any pigment-concentrating effect of RPCH [64] without further evidence to support it. The peptides in inserts and crustaceans can only be a reference but not solid evidence for barnacle research.

Currently, bioinformatic methods, including in silico genome and transcriptome mining, are widely employed for the discovery of natural products from barnacles and have proved to be successful and effective [23,47]. The identification of neuropeptides in barnacles primarily relies on transcriptomics data; however, visual studies on neuropeptides can provide more comprehensive insights. Kalke et al. imaged the nervous system of barnacles in different stages by immunolabelling and observed their variation [49]. Integrating imaging techniques with bioinformatic tools could be a promising strategy to elucidate the underlying mechanism and validate the functional process of specific neuropeptides. Furthermore, the identification of unique neuropeptide structures in barnacles, which could potentially modulate their functional efficiency, may provide additional evidence for neuroscientific research.

Integrating the bioinformatic method, imaging method, validation experiment, and extraction method can deepen our understanding of peptides in barnacles and their functions and enable the exploration of more antifouling strategies (Table 1).

### 3.2. Settlement Peptide—Adsorption Step in Biofouling

Numerous environmental factors, such as color, olfaction, biofilm, planktonic micro-community, and chemicals, are considered significant cues for biofouling settlements. Settlement-inducing protein pheromones and barnacles with water-borne protein complexes [25] were found to play a vital role in the settlement process both separately and jointly, and the composition, especially the ratio, was closely related to the habitat chosen, adult–adult interaction, and varied between different species of barnacles [66]. Individually, settlement-inducing protein pheromones, a contact pheromone, could induce larvae settlements and function as an adhesive [67]. Tegtmeyer used to claim that peptides with a basic carboxy-terminal amino acid and either a neutral or a basic amino-terminal amino acid could enhance their settlement without further proof [68]. There is little to the structure of the barnacle peptide recorded for a structure–activity relationship that the chemical modification puts forward slowly.

Barnacles are sensitive to sound, color, and olfaction during the exploration stage, and the cyprid relies on sensory mechanoreceptive and chemoreceptive signals to decide the fouling status. Sound can be a neglected factor during the settlement, especially at the cyprid, the nauplius VI stage, and late stage (juvenile and adult), working as an important communication method [36]. Five octopamine receptor subtypes were expressed in the cyprids and further related to the settlement process [69]. Barnacles are supposed to release antibacterial peptides to kill bacteria on the surface before their settlement [70]. The interaction between the bacteria and barnacles, especially the community structure of the biofilm, has been proven to affect the settlement choice of barnacles, especially during the period from cypris larvae to sessile juveniles [44]. The expression of arginine kinase, a primary enzyme related to cellular energy metabolism, varies according to environmental conditions, such as oxidation stress hypoxia and energetic stress regulated by the P38 MARK and NO/cGMP pathway [71,72]. Besides the environmental factors, it is believed that there are active interactions of larvae–adult and adult–adult through the chemical signal, and this also works intraspecifically [73]. There were few exact peptides identified in barnacles that self-assembled monolayers were applied to when exploring antifouling peptides. Ederth’s team designed five peptides and tested the self-assembled and antifouling ability of these peptides [74].

Though there are few peptides identified exactly from barnacle cement, the sequence result from tandem mass spectrometry found peptides similar to bovine trypsin and human transglutaminase, such as XIII A1 peptides [75], which showed that there are more remain unexplored. However, this exploring method lacked further validation, and the extraction method limited the identification of exact functional peptides: a general gap for natural product discovery. Though people have found many strains of algae that could inhibit the settlement of barnacles, and some peptides have been proven to work as signal function, no exact peptides have been identified, while many chemicals have been extracted and identified [76].

Nitric oxide is an important signal transduction molecule and has been reported to negatively affect the larval settlement in various fouling organisms, including gastropods, ascidians, sea urchins, annelids, and barnacles [77]. Current studies have found that nitric oxide signaling may control the overall settlement process in barnacles, and its regulation is mainly conducted via guanylyl cyclase and cyclic guanosine monophosphate (cyclic GMP, cGMP) [39]. In addition, nutrients have been proven to have a close connection with the settlement of barnacles. For many years, people have found that the natural inhibitor cues released by algae and coral could avoid sessile fouling [78]. Based on this connection, Jasmin designed a field experiment and found that barnacles preferred nutrient-enriched biofilm, which differed from other fouling communities [79]. Researchers have shed light on many different physiological processes. However, the reason for these phenomena on the molecular level remains unknown and worth further exploration.

### 3.3. Adhesive and Chemosensory Peptides—The Attachment Strategies of Barnacles

Barnacles have developed remarkable attachment strategies to undergo adhesion without any degradation, even decades after death [80]. This characteristic makes barnacles the main biofouling organism in the marine environment [36]. This is a dramatic and complicated process, including the adsorption process, water displacement, adhesion, and cohesion process. Researchers have devoted a lot of effort to these processes of interest [81]. Based on this fantastic characteristic, researchers are interested in exploring the biochemistry process and have applied it in medical use [81]. Barnacle cement is not a uniform structure, and there are two other interfacial types near the bulk cement. 19kDa peptides were discovered to bind to interfacial cement but did not gain much attention because of their insoluble nature [82]. Over 94% composition of barnacle cement is a multi-protein complex, and numerous short barnacle cement-derived peptides are dominant, but their function remains ambiguous [83]. Moreover, the composition of cement can change in accordance with the site and life stages. Dany’s studies have proved this in *Policipes pollicipes* [31], where the gland has more identified peptide sequences than cement, dominated by the muscle, cytoskeleton, and other uncharacterized peptides. The polymerization mechanism of barnacle cement raised an interest in underwater adhesive studies. Polypeptides, a chain of amino acids bound together via covalent peptide bonds, were commonly found in barnacle cement [84]. Raman’s team studied peptides Bp1(VPPPCD) and Bp2 (KLDLLTDG), which have both hydrophobic and charged groups, and revealed that the physical structure of peptides could affect adhesion strength [85]. The tight attachment of barnacles is the key challenge for antifouling, where weakening the attachment of barnacles could be a new strategy.

Since more methods, such as self-assembled monolayer techniques, have been successfully used to study the molecular interaction mechanisms of barnacle peptides, more cement peptides need to be explored and deeply learned, which could give more inspiration to the application of barnacle cement for medical use or industrial material. So et al. [86] found that the repeated simple domain and charged domain lie on the full-length 19 kDa protein. Based on this discovery, two biomimetic peptides were designed: one with charged domains and low-complexity domains, which could induce self-assembly and self-organization into nanofibrils, while the other type only had the function of self-assembly. The cement protein Balcp19k was found to form entangled nanofibrils and nanorings under acidic and low-ionic-strength conditions [87]. The detailed designed peptides and barnacle-derived peptides are listed in Table 2.

Self-assembling peptides exhibit specific physicochemical and biochemical activities as well as a potential in antifouling. The direct application of the barnacle-derived peptides is challenging due to their low yield, while self-assembling peptides broaden the scope of potential application. These peptides exhibit good effects on microfouling and the settlement process of barnacles. Researchers have attempted to synthesize key recombinant barnacle cement or barnacle-inspired peptides. These findings show the feasibility and great application potential of self-assembling peptides inspired by barnacles in antifouling and other related fields. It is also of great importance to reveal how barnacles achieve strong adhesion via self-assembling, and this is also a good strategy to block the biofouling process by inhibiting the adhesion.

## 4. Conclusions

Barnacles, globally renowned marine fouling organisms, possess limited natural predators. Researchers have dedicated over a century to studying barnacles, with a specific emphasis on antifouling mechanisms and cement application. However, the physiological activities and in vivo chemical reactions of barnacles remain largely enigmatic. Although only a restricted number of barnacle-derived peptides have been discovered and characterized thus far, they serve as an inspirational foundation for novel antifouling strategies.

The intricate fouling process of barnacles is intricately regulated by neuropeptides, settlement peptides, and chemosensory peptides. The pivotal role of these peptides in the biofouling process renders them promising targets for antifouling interventions. Furthermore, an enhanced comprehension of barnacle attachment mechanisms could facilitate researchers in exploring a wide range of natural products with diverse activities, thereby expanding the potential applications of barnacle-derived peptides. Despite the significant progress that has been made in barnacle research, numerous knowledge gaps still exist that necessitate further investigation to elucidate their precise underlying mechanisms. Most conclusions and discovered peptides in barnacle research are speculative and uncertain. Proteins and peptides in barnacles have been identified using bioinformatic methods; however, their validation was insufficient. Researchers are gradually recognizing the crux of the matter and are striving to enhance these methodologies. This review aims to provide a comprehensive overview of the advancements made in barnacle-derived peptides, demonstrating the untapped potential of peptides in antifouling applications and expects to inspire further innovation in this field.

## Figures and Tables

**Table 1:** Neuropeptide related to the biofouling process in barnacles.

Peptide	Related Functions	Amino Acids Sequence	References
Pignent depersing hormone (PDH)	Regular color change and sensing	NSELINSILGLPKVMNDA	[57]
Crustacean Cardioactive peptide (CCAP)	Control the ecdysis and stomatogastric behavior	PFCNAFTGC	[52]
A-type allatostatin	Related to the nutrient state such as lipids usage regular metabolism	Conserved pentapeptide C-terminal sequence Y/F-X-F-G-L/lamide	[60]
B-type allatostatin	Muscle control, metabolism regulation, feeding, sleeping	With conserved W(X0) amide at the C-terminus	[61]
C-type allatostatin	Modulating the neuromuscular system	Characterized by a non-amidated c-terminal pentapeptide PISCF motif	[7]
Calcitonin-like diuretic hormone-isoform A	Maintain ionic homeostasis of hemolymph during barnacle development	GFDFGLGRGFSASQAAKHKMGLEAAEFPSGPa	
SIFamide	Transmitting neural signals and detecting exogenic cues in the settlement processes	MGSRCAVRRVAAVLVVALVAMALLAPLTEAGYRKPTFNGSIFGKRAAAAAEAEAAQGLARMCAAAYTVCGFPAE	
Tachykinin-relate peptide (TRPs)	Feeding-related behavior: food intake and digestion-related functions	Phe–Xaa1–(Gly/Ala)–Xaa2–Arg–NH2 for most TKRPs or Phe–Xaa1–Xaa2–Xaa3–Arg–NH2 for natalisins	
The prothoracicotropic hormone (PTTH)	Promote larval development	unsigned	[23]
FMRFamide-like peptides	Neurotransmitter in the central nervous system and gut Neurosecretory role in the sensory function of frontal filament	A group of peptides with N-terminally extended Phe-Met-Arg-Phe-NH2	[57]

Some sequences were referred from NCBI (2022) [65].

**Table 2:** Peptide fragments from barnacles and their modifier homologues related to adhesive and chemosensation.

Peptide	Amino Acids Sequence	Amide Length (aa)	(Inspired) Source	References
cMr20-S5	Ac-SKLPCNDEHPCYRKEGGVVSCDCK	24	Mrcp- 20k	[88]
cMr20-S6	Ac-KTITCNEDHPCYHSYEEDGVTKSDCDCE	28		
Bp1	GSGSVPPPCD	10	Interfacial cement	[85]
Bp2	GSKLDLLTDG	10		
BCP1	QTGYTRGGAAVSSTGATQGAGS	22	Full-length 19 kDa protein	[86]
BCP1C	QTGYTRGGAAVSSTGATQGAGSLDLAIDGPGGFKARSK	38		
BCP2	AVGNSGVSGSGVSIGDSGFRQKTQT	25		
BCP2C	AVGNSGVSGSGVSIGDSGFRQKTQTNSEAGSKGTKRA	37		
BCP3	TGTQGKGITSGEAVANQKAGAEGG	24		
BCP3C	TGTQGKGITSGEAVANQKAGAEGGAQRVEAVKYVESDGKNLYKVEKVD	48		
BCP4	GTSSSGHKASSSGPGRFITSN	21		
BCP4C	GTSSSGHKASSSGPGRFITSNEVGTEIKLTTPELD	35		
mutBCP1	QTGYTRGGAAVSSTGATQCAGS	22		
P1	VPPPSDLSIKSKLKQV	16	Barnacle cp19k	[87]
P2	GATKGNAAVTTKGTTSGS	18		
P3	GVVKSVVRTPTSVEKK	16		
P4	KAAVGDTGLSAVSASADNG	19		
P5	LFKNLGKATTEVKTTKDGTKVKTK	24		
P6	TAGKGKTGGTATTLQIADANGG	22		
P7	VSEKSLKLDLLTDGLKFVKVTEKKQ	25		
P8	GTATSSSGHKASGVGHS	17		
P9	VFKVLNEAETELELKGL	17		
R1-3	RRKYSGILGDLIQVAVIRYY	20	cp52k	[89]

## Data Availability

The data presented in this review are available in the main text.
